# Analysis of 55 cases of adenomatoid odontogenic tumor in an Indian population and review of literature

**DOI:** 10.4317/medoral.24977

**Published:** 2021-12-07

**Authors:** Shivani P Bansal, Sana Shaikh, Ankita Satish Arvandekar, Sonal S Dhanawade, Rajiv S Desai

**Affiliations:** 1 M.D.S., Professor (Additional). Department of Oral Pathology, Nair Hospital Dental College, Mumbai, India; 2 M.D.S., Ex-post Graduate Student. Department of Oral Pathology, Nair Hospital Dental College, Mumbai, India; 3 B.D.S., Post graduate student. Department of Oral Pathology, Nair Hospital Dental College, Mumbai, India; 4 M.D.S., Professor and Head. Department of Oral Pathology, Nair Hospital Dental College, Mumbai, India

## Abstract

**Background:**

This study reviews the demographic, clinical and radiographic features of adenomatoid odontogenic tumor(AOT) diagnosed in an Indian population over 50 years and also evaluate and compare follicular AOT(F-AOT) and extra-follicular AOT(EF-AOT).

**Material and Methods:**

55 diagnosed cases of AOT from 1971-2020 were studied retrospectively. The data regarding the age, sex, location, variant of AOT, duration, clinical features, radiographic appearance, treatment and recurrence were collected and analysed.

**Results:**

Of the 722 odontogenic tumors diagnosed, 7.6% were AOTs with higher prevalence of extra-follicular (67.3%) than follicular (32.7%) variant. All the tumors were intraosseous with a marked predilection for maxilla over mandible, ratio 2:1. The patients mean age was 19.8 years with slightly higher female predilection (male:female ratio - 1:1.5). The anterior region (76.4%) was more frequently affected and entire quadrant was involved in 21.8% cases. Clinically, asymptomatic, slow-growing swelling was seen in 81.8% cases with duration of 15 days to 10 years. Radiographically, AOT appeared as well-corticated radiolucent lesion. Canine was the most commonly impacted tooth. Recurrence was seen in 3 cases.

**Conclusions:**

Interestingly, in this series extra-follicular was twice more common than follicular AOT. Few cases involved the entire quadrant or crossed the midline of either jaws.

** Key words:**Adenomatoid odontogenic tumor (AOT), follicular AOT, extrafollicular AOT, Indian population, odontogenic tumor.

## Introduction

Adenomatoid odontogenic tumor (AOT) is regarded as a benign neoplasm of odontogenic epithelial origin showing characteristic duct-like structures. AOT represents only 0.1% of all tumor and cysts of the jaw and account for 2.2% to 7.1% of all odontogenic tumors, marking them as fourth or fifth most common odontogenic tumor (1). However, based on a multicentric study, the relative frequency of occurrence of AOT was reported from 0.6 to 38.5% based on geographic location (2).

In 1903, AOT was first described by Nakayama as ‘cystic epithelial tumor’, while Harbitz reported the first European case of AOT in 1915 (3,4). The term adenomatoid odontogenic tumor was coined, in 1969, by Philipsen and Birn and was adopted by World Health Organization (WHO) Classification of Odontogenic Tumors in 1971 (5,6). AOT is regarded as a true benign, non-aggressive, non-invasive neoplasm with limited growth potential but few authors categorize them as hamartomas (1).

AOT is present in a fairly consistent manner, as a result of which it has come to be regarded as a “tumor of two-thirds”, i.e. two-thirds occur in female patients, two-thirds occur in the second decade of life, two-thirds develop in the anterior region of the maxilla, two-thirds are superimposed on dentigerous cysts, and in two-thirds of cases associated unerupted teeth are permanent canines. In addition, two-thirds of cases show scattered radio-opacities within the lesion (3). Depending on its location, AOT is classified as 1. Central AOT:- (a) Follicular type (F-AOT) - occurs centrally as a well-defined radiolucency which is associated with crown of an impacted tooth, (b) Extra follicular type (EF-AOT) - occurs centrally as a well-defined radiolucency and is not associated with an impacted tooth and 2. Peripheral AOT (P-AOT) - occurs in the soft tissue overlying tooth bearing area or alveolar mucosa in the jaws or represents erupted intraosseous peripheral AOT and rarely producing any radiographical changes (2,4,7-9).

AOT is derived from odontogenic epithelium of the dental lamina complex or its cellular remnants (4). Microscopically, AOT is composed of solid nodules of polygonal, cuboidal or spindle-shaped odontogenic epithelial cells. These cells form nests, duct-like spaces, rosette-like structures and strands with a trabecular and cribriform pattern, in a mature connective tissue stroma, surrounded by a fibrous capsule. Intercellular eosinophilic amorphous material and varying amount of calcified material are present in most lesions (1,7).

Since there is a paucity of large case series regarding AOT from south-east Asia especially India, the present study was conducted to evaluate the relative frequency, clinical and radiological features of AOT in an Indian population and compare with the world literature.

## Material and Methods

All the diagnosed cases of AOT, over 50 years period (1971-2020) at the Oral Pathology Department, Nair Hospital Dental College, India, were retrieved. The Institutional Ethics Committee of Nair Hospital Dental College (IEC-NHDC) approved the study (EC-35/DOMR-04-ND/2016). It was designed according to the principles manifested in the Declaration of Helsinki and consistent with the guidelines of Good Clinical Practice given by International Conference of Harmonization (ICH-GCP). The haematoxylin and eosin (H&E) stained slides were re-examined to confirm the diagnosis by SB and SS. The histopathologic diagnosis of AOT was made according to the WHO 2017 classification of odontogenic tumors (10). The data regarding patient age, sex, location of lesion, duration, clinical features, radiographic appearance, treatment, follow-up and recurrence was recorded. Regarding the site of occurrence, the jaw was divided into anterior region (from incisor to canine) and posterior region (premolars and molars) respectively.

## Results

From 1971-2020, 722 odontogenic tumors were reported of which 55 were diagnosed as AOT (7.6%), Table 1, Table 2 and Table 3 gives demographic data and decade-wise distribution of 55 cases of AOT. The highest prevalence of AOT was seen in the second decade of life (33 cases, 60%) with an age range between 10 and 50 years (mean age=19.8 years). All except, one case occurred at the age of 10 and two cases at the age of 50 years. The mean age for F-AOT and EF-AOT was 18.6 years and 20.9 years respectively with an age range of 14-24 years in F-AOT and 10-50 years for EF-AOT. There were 22 males and 33 females, with a male:female ratio of 1:1.5. The mean age of males (21.2 years) was slightly higher than females (19.4 years).

**Figure 1:**
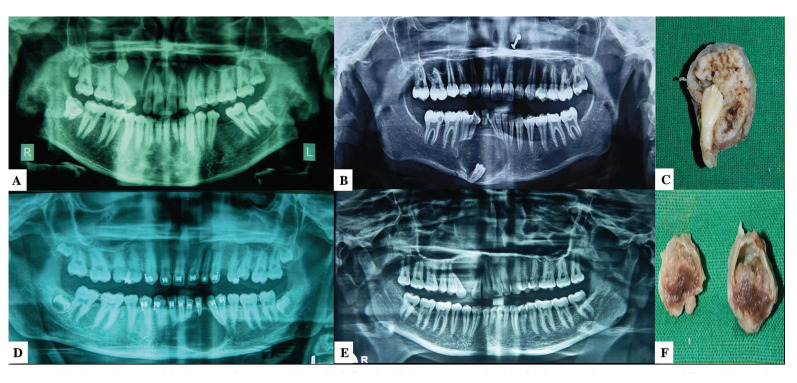
Follicular adenomatoid odontogenic tumor: (A) Well-defined radiolucency associated with impacted permanent maxillary right canine. (B) Well-defined radiolucency associated with impacted permanent mandibular canine. (C) Gross specimen of tumor with impacted tooth. Extra-follicular adenomatoid odontogenic tumor: (D) Well-defined radiolucency in mandibular left incisor and canine region. (E) Well-defined radiolucency in maxillary right incisor and canine region. (F) Gross specimen of tumor.

55 cases of AOT included 37 cases (67.3%) of EF-AOT (maxilla- 26, mandible- 11) and 18 cases (32.7%) of F-AOT (maxilla-11, mandible-7) (Fig. 1). Not a single case of P-AOT was observed in our study. There was a noTable predilection for the maxilla (37 cases, 67.3%) over the mandible (18 cases, 32.7%) with a ratio of 2:1. The anterior region (42 cases, 76.4%) was more frequently involved as compared to the posterior region of the either jaws (1 case, 1.8%). 6 cases (10.9%) in the anterior region of the jaws showed midline crossing, 1 in the maxilla and 5 in the mandible. The remaining 12 cases (21.8%) included 7 in the maxilla and 5 in the mandible involving the entire quadrant from incisor to molars (Fig. 1). One case in maxilla extended till the tuberosity region. Grossly, the size of the tumor ranged from 1.5cm to 4.5cm (mean-2.5cm).

**Figure 2:**
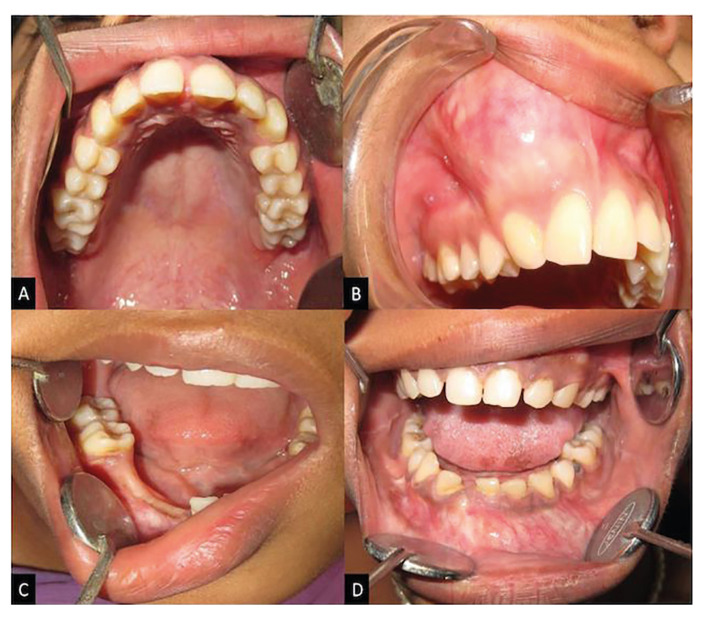
Intraoral photograph of maxilla showing: A) No significant changes B) Diffuse, firm, smoothly surfaced swelling in the anterior right maxillary region with missing permanent maxillary right canine. Intraoral photograph of mandible: C) No significant changes in the right mandibular region. D) Diffuse swelling causing obliteration of labial vestibule with missing permanent mandibular right central incisor.

Clinically asymptomatic, slow-growing swelling was evident in 81.8% (n=45) cases, which varied from 15 days to 10 years (Fig. 2).

**Table 1:**
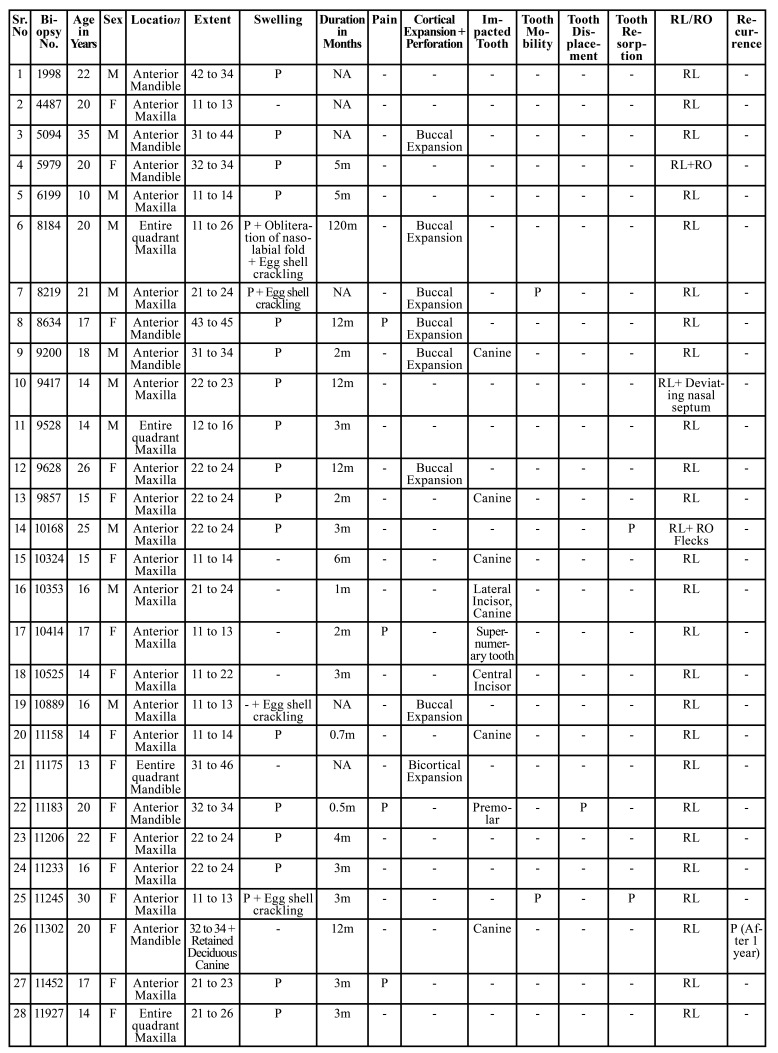
Clinical and radiographic data of 55 cases of AOT.

**Table 1 cont.**
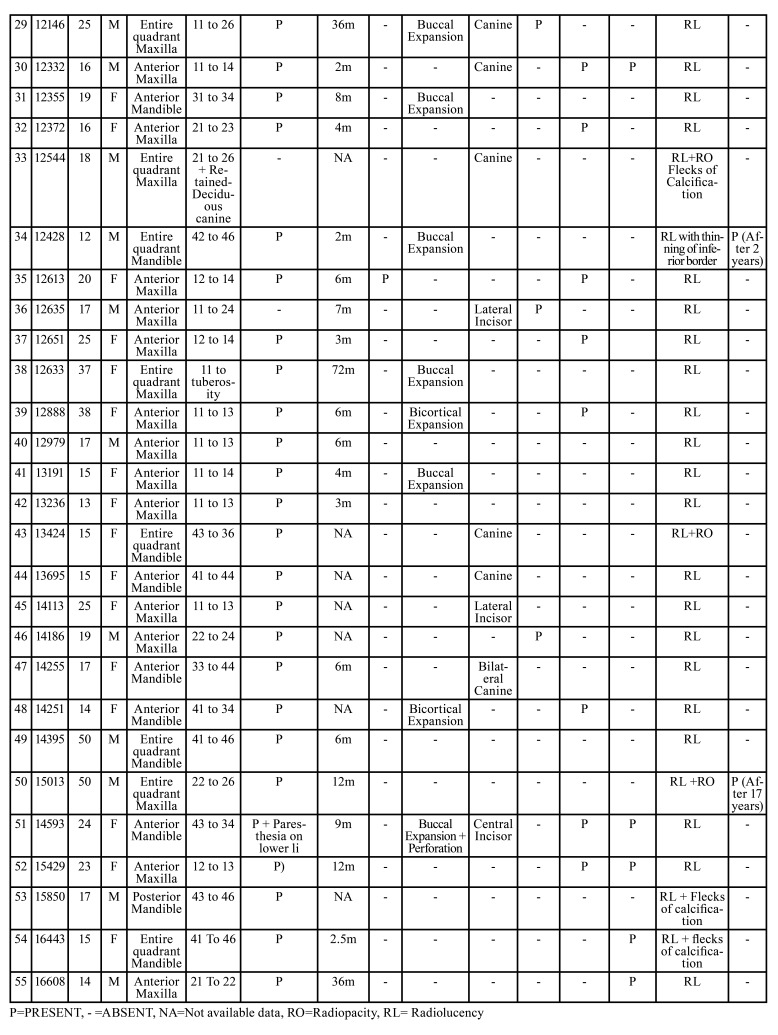
Clinical and radiographic data of 55 cases of AOT.

**Table 2:**
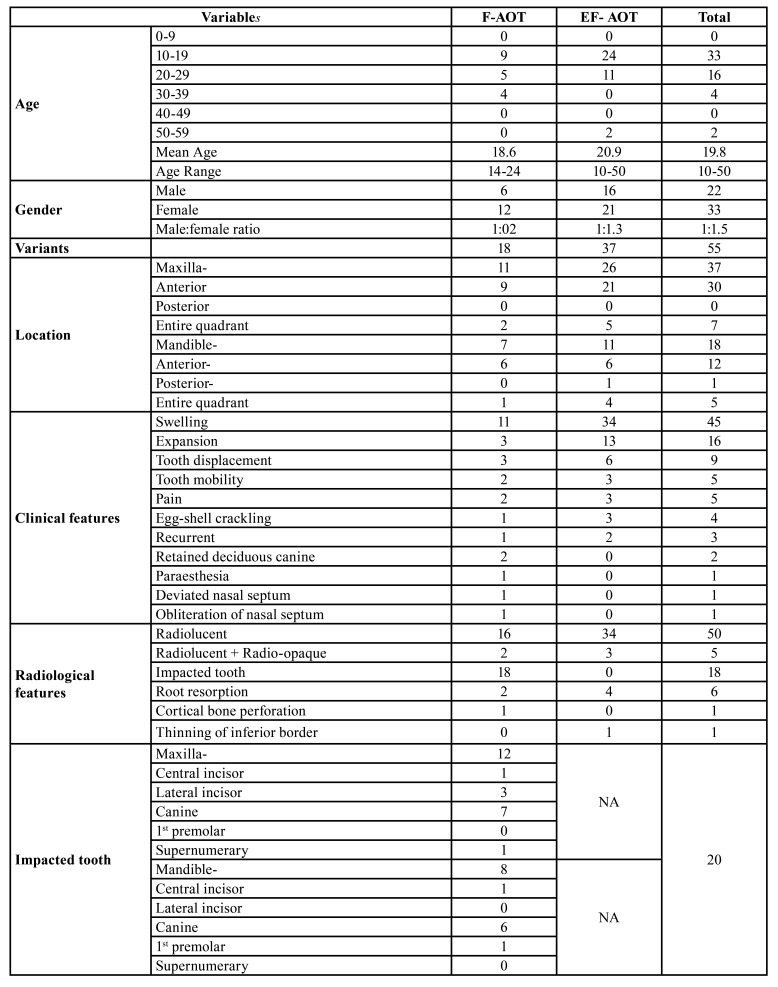
Distribution of 55 cases of AOT.

**Table 3:**
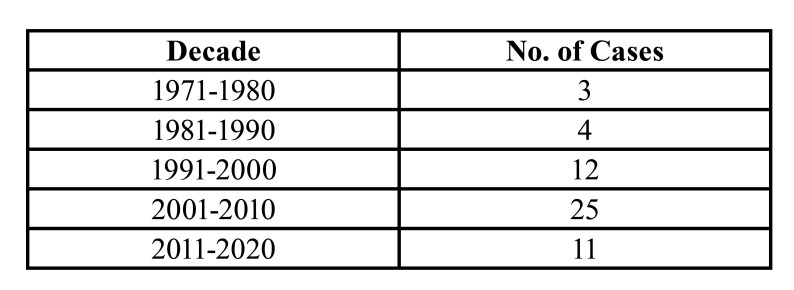
Decade-wise distribution of AOT cases.

Intraorally, cortical expansion (n-16, 29.1%) was the most common finding followed by tooth displacement (n-9, 16.4%), tooth mobility (n=5, 9.1%) and pain (n=5, 9.1%). The bicortical expansion was seen in 3 cases (18.7%) whereas remaining 13 cases (81.3%) showed only labial/buccal cortical expansion. In isolated cases, egg-shell crackling (n=3, 5.3%), retained deciduous canine (n=2, 3.6%), deviated nasal septum (n=1, 1.8%), obliteration of nasolabial fold (n=1, 1.8%) and paraesthesia (n=1, 1.8%) was observed.

The radiographic examination revealed unilocular radiolucencies with well-corticated borders in 50 cases (90.9%) and mixed radio-opaque/ radiolucent image in 5 cases (9.1%). Root resorption (n=5, 9.1%), thinning of inferior border (n=1, 1.8%) and perforation (n=1, 1.8%) was also noted. In the maxilla permanent canine (n=7, 54.5%) was the most commonly impacted tooth followed by permanent lateral incisor (n=3, 27.3%) and central incisor (n=1, 9.1%). The most commonly impacted tooth in mandible was the permanent canine (n=6, 75%) followed by permanent central incisor (n=1, 12.5%) and first premolar (n=1, 12.5%). From 18 cases of follicular AOT, 16 cases showed single impacted tooth whereas 2 cases showed bilateral permanent canine impaction and one case showed permanent lateral incisor and canine impacted. A case of an impacted supernumerary tooth was also seen in our study. No impacted deciduous teeth and impacted permanent molars were involved in our study.

**Figure 3:**
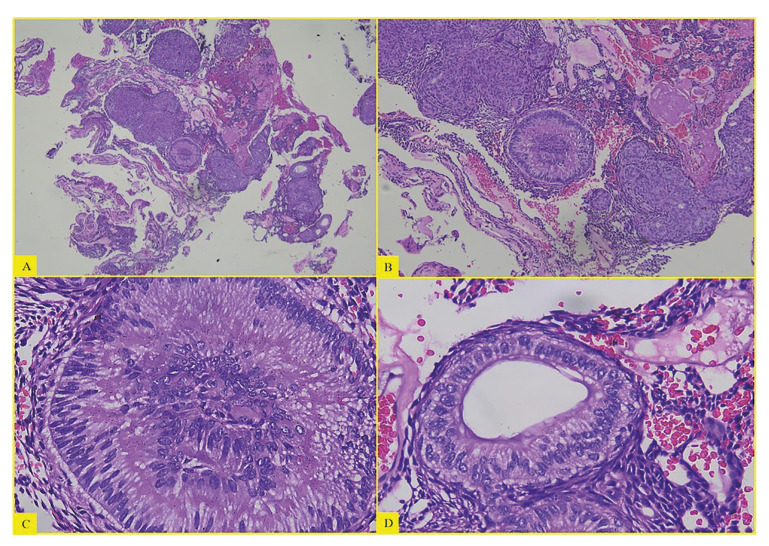
(A) Cellular multinodular proliferation of cells in the form of nests and rosettes. (H and E, 4X). (B) Solid areas of cells in the form of whorls interspersed with areas of calcification. (H and E, 10X). (C) Inset showing the characteristic rosette like structure (H and E, 40X). (D) Duct-like pattern lined by cuboidal cells (H and E, 40X).

Histological analysis revealed the typical findings of AOT in all cases, with varying-sized nodules and rosette-like structures composed by spindle shaped to cuboidal epithelial cells with intercellular droplets of eosinophilic material scattered in some areas. The duct-like spaces were lined with a single row of cuboidal epithelial cells. Interlacing strands of epithelium with one to two cells in thickness forming a trabecular or cribriform conFiguration were seen at the periphery of the more solid areas. Dystrophic calcifications in varying amounts and in different forms were encountered in most AOTs within the lumina of the ductlike structures, scattered among epithelial masses or in the stroma (Fig. 3).

All the patients were treated with enucleation. Recurrence was seen in 3 cases of AOT. A case of F-AOT showed recurrence after 1 year and 2 cases of EF-AOT showed recurrence after 17 years and 2 years respectively.

## Discussion

AOT is a rare benign epithelial odontogenic tumor and accounts for less than 5% of odontogenic tumors (1). In a worldwide literature survey by Philipsen et.al. the relative frequency of AOTs was found to be higher ranging from 0.6%-38.5% based on the geographical location. The distribution of AOTs in the different parts of the world is: Africa 1-38.5%, Asia 1-16%, South America 4-7%, North America 2-7%, Middle East 2-4% and Europe 1-4% (2). In the present study, AOT represented 7.6% of all odontogenic tumors a range well within those seen in the other parts of the world. However, when compared with Asian countries we observed a slightly higher relative frequency than those reported from Thailand, Hong Kong, Taiwan, Japan, Malaysia but slightly lower than those reported from China and Sri Lanka (Table 4) (2,11-17).

**Table 4:**
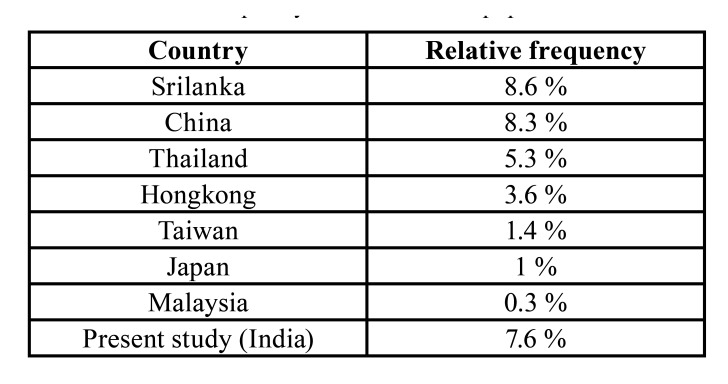
Relative frequency of AOT in Asian population.

The 55 cases in our series had an average age of 19.8 years with the peak incidence in the second and third decade of life, which is in accordance with the literature (1,2,12-19). Although, it is uncommon for AOT to occur in patients older than 30 we found two patients in 50 years of age group. We also observed that the mean age for EF-AOT (20.9 years) was slightly more than F-AOT (18.6 years). The male to female ratio for Asians, non-Asians and Africans is 1:2.3, 1:1.4, and 1:2.5 respectively (7,19). In our series, the male to female ratio was 1:1.5 which is lower than the global ratio of 1:1.9 and much lower than those reported in the Asian population. Published Indian study has a lower male to female ratio of 1:1.1 as compared to our findings (2,19,20). Ours is the second study from the Indian subcontinent after Chattopadhyay A, having a larger sample size indicating a regional variation in gender distribution (20).

All the cases of AOT were of intraosseous type. The present study showed marked predilection for the maxilla while Siar and Ng and Fernandez *et al* reported slight predilection for the mandible (15,21). The canine was the most common impacted tooth, which is consistent with the literature (1,2). Leon *et al* in the immunohistochemical and ultrastructural study of AOT has proposed plausible origin of AOT from the reduced dental epithelium which could partly explain its prevailing association with the crown of an impacted tooth (9). The anterior region of the jaw was the most representing site as suggested by various studies (1,2) The present study found 10.9% (n=6) of AOT crossing the midline against 8% (n=16/201) as reported in the literature (18). Surprisingly, we also observed AOTs extending from incisor to molars involving the entire quadrant of both maxilla and mandible, which has not been reported so far in an Asian population (11,13,15,16). Mohamed et. al found 61% cases affecting the entire quadrant in a black South African population (19). Another interesting finding of our series was strikingly higher prevalence of EF-AOT(67.3%) than F-AOT(32.7%) in accordance with another Indian study by Chattopadhyay A, unlike the literature survey that reports all (Africa study) or majority of cases as follicular variant (1,2,19,22). Maxilla was the common site of occurrence for both F-AOT and EF-AOT, unlike Arotiba *et al* and Adisa *et al* who found EF-AOT more common in the mandible (8,22). Roza ALOC *et al*. recently concluded that the posterior regions of the gnathic bones were affected and most of the tumors demonstrated larger sizes (23).

**Figure 4:**
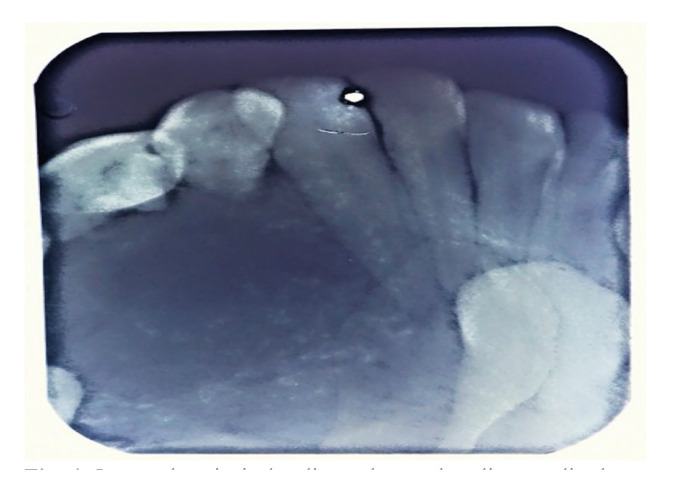
Intraoral periapical radiograph reveals solitary radio-dense mass with internal calcifications enclosing the impacted 34, mesially displacing the roots of 31 and 32.

Radiographically, orthopantomograph shows intraosseous AOT as a well-defined unilocular radiolucency exhibiting the presence of calcified material in two-third cases whereas the current study showed the presence of calcified material in 5 cases only (Fig. 4). It has been suggested that intra-oral periapical radiographs are better suited for showing intralesional discrete calcified deposits than panoramic radiography (1,7).

AOT is characterized by an asymptomatic, slow but progressive growth. Tooth displacement and mobility can be encountered but root resorption is unusual. Bone expansion is a common finding, while perforation of the cortical plate is rare.

Our study showed slow growing, asymptomatic swelling exhibiting bony expansion as the most common presenting symptom. Recurrence was observed in 3 cases (5.5%) during a long term follow up, which could be due to a development of a novel tumor or an incomplete excision of the primary tumor. The recurrent tumors shared the same site of occurrence and had histological features similar to the primary tumor. Chrcanovic and Gomez in their review highlighted a single case of recurrence in 1500 cases and pointed that there are some cases of recurrence in the literature with uncertain histopathology or may represent a residual tumor after incomplete removal. They suggested the limited period of follow-up of not more than 39 months could have led to miscalculation of the recurrence rate in AOTs (18). We observed more cases of EF-AOT exhibiting involvement of the entire quadrant and recurrence rate than F-AOT.

## Conclusions

The noteworthy features of our largest case series from India were, i) EF-AOT was twice more common than F-AOT, unlike that reported in the global literature, ii) few cases involved the entire quadrant or crossed the midline of either jaws, iii) Patients with AOT should be kept under regular follow up as we had cases of recurrence during long term follow-up.
